# PCL/Sodium-Alginate Based 3D-Printed Dual Drug Delivery System with Antibacterial Activity for Osteomyelitis Therapy

**DOI:** 10.3390/gels8030163

**Published:** 2022-03-05

**Authors:** Ji-Hyun Lee, Jung-Kyu Park, Kuk-Hui Son, Jin-Woo Lee

**Affiliations:** 1 Department of Molecular Medicine, College of Medicine, Gachon University, 155, Gaetbeol-ro, Yeonsu-ku, Incheon 21999, Korea; totoro218@hanmail.net; 2 Department of Health Sciences and Technology, GAIHST, Gachon University, 155, Gaetbeol-ro, Yeonsu-ku, Incheon 21999, Korea; pj920903@naver.com; 3 Department Thoracic and Cardiovascular Surgery, Gachon University Gil Medical Center, College of Medicine, Gachon University, 21, Namdong-daero 774 beon-gil, Namdong-gu, Incheon 21565, Korea

**Keywords:** osteomyelitis, biofilm, 3D printing, encapsulation, dual drug delivery system

## Abstract

Chronic osteomyelitis is mostly caused by bacteria such as *S. aureus*, and is often treated with oral antibiotics or injections to suppress the bacteria. In severe cases, however, surgical treatment using antibiotic beads and metal supports may be required. In these surgeries, bacterial attachment to the metal may lead to biofilm formation and reduce antibiotics’ penetration to the bacteria. Reoperation must be performed to prevent bacterial inflammatory reactions and antibiotic resistance. Thus, in this study, we developed a dual-drug-releasing PCL/sodium-alginate-based 3D-printed scaffold to effectively treat osteomyelitis by removing the biofilm. We proposed an antibiotic-loaded biodegradable polymer scaffold using 3D printing, which was encapsulated by a second antibiotic-containing hydrogel. Then, we successfully established a dual-drug-based scaffold that consisted of a cefazolin (CFZ)-containing polycaprolactone 3D scaffold and a rifampicin (RFP)-loaded alginate hydrogel encapsulating the 3D scaffold. Our scaffold showed a synergistic effect, whereby biofilm formation was inhibited by RFP, which is an external drug, and bacterial activity was inhibited by CFZ, which is an internal drug that increases antibacterial activity. We also confirmed that the dual-drug-based scaffold did not affect the proliferation of human osteoblasts. Our findings suggest that this dual drug delivery system may serve as a new therapeutic treatment for osteomyelitis that overcomes the limitations of individual drugs.

## 1. Introduction

*Staphylococcus* constitutes up to two-thirds of the total pathogens in orthopedic implant infections, and is the major causative agent of osteomyelitis, causing inflammatory destruction of bones [1]. When *S. aureus* is identified as the causative strain, a therapeutic antimicrobial agent from among several types of antibiotics—including nafcillin, cefazolin (CFZ), aminopenicillin, vancomycin, and rifampicin (RFP)—is selected based on the results of antibacterial sensitivity testing [2,3,4]. Due to the increasing frequency of *S. aureus* infections in osteomyelitis, the rapid development and display of multiple antibiotic resistance, and the tendency to progress from acute infections to persistent, chronic, and recurrent infections, this pathogen continues to receive considerable attention [5].

Treatment of these infections is complicated by the formation of bacterial biofilms, which are implicated in nearly all cases of osteomyelitis [5]. A biofilm is defined as a microbially derived sessile community, typified by cells attached to a substratum. Biofilms impede the ability of immune cells to reach bacteria, and reduce the penetration and efficacy of antibiotics [6]. Patients with implanted medical devices, such as prostheses or catheters, are at risk of biofilm infections [7,8]. *Staphylococcus* spp. can produce a multilayered biofilm embedded within a glycocalyx, also known as a slime layer. The glycocalyx develops on devitalized tissue and bone or implanted devices, and increases susceptibility to infection [9]. Bacterial biofilm infections are problematic because sessile bacteria can withstand host immune responses and are drastically more resistant to antibiotics [5]. As a result, the eradication of biofilms requires higher concentrations of antibiotics, increasing the likelihood of multidrug resistance.

Our 3D printing approach has notable advantages over conventional drug delivery systems, such as customized and flexible drug design capabilities in choosing the desired dose, shape, and size for patient needs [10]. Polycaprolactone (PCL) is one of the most common materials used in fabricating scaffolds for tissue regeneration [11]. This polymer is a Food and Drug Administration (FDA)-approved linear polyester with good biocompatibility, slow degradation rate, fewer acidic breakdown products in comparison to other polyesters, and the potential for loadbearing applications [12,13]; in addition, it has a low melting point (59–64 °C) and exceptional blend compatibility with other synthetic polymers [14]. We previously reported an antibiotic-loaded scaffold fabricated using a 3D printing technique developed via a cold fabrication process that was suitable for thermosensitive antibiotics [12]. CFZ, with its *S. aureus* and limited Gram-negative coverage, is widely used by the orthopedic community to treat adult osteomyelitis [15]. However, while CFZ—a cephalosporin—has excellent in vitro activity against *S. aureus*, it is a heat-labile antibiotic and, thus, has limitations in osteomyelitis treatment [16].

Alginates are linear, water-soluble, high-swelling natural anionic polysaccharides, and are acknowledged to have excellent biocompatibility [17]. These biopolymers consist of 1,4-linked β-D-mannuronic acid (M) and 1,4 α-L-guluronic acid (G) units [18,19]. The most used alginate types in wound-healing studies are calcium and sodium alginates. By tuning, the alginate concentration viscosity and other physicochemical properties are influenced. Moreover, the speed and the drugs’ absorption rate depend on the wound pH and drug type, but also on the solubility of the alginate salt. Due to their properties, alginates are the most used biomaterials for wound-dressing products [17,20].

RFP has excellent oral bioavailability (70–90%) and strong anti-staphylococcal activity [21]. The efficacy of RFP against *Staphylococcus* infection in bones and joints has been proven in many animal models [22,23]. Biofilm penetration and antibiotic activation are known to be possible [21]. Therefore, bones, joints, and particularly implants are important sites for antibiotic action. An effective way to prevent biofilm formation is to coat antibacterial agents such as metal derivatives, polyammonium salts, and antibiotics on the substrate surface [7,24]. Hydrogels are commonly used to deliver wound drugs because they remove excess exudates, promote rapid wound healing, are easily removed without trauma, and have excellent biocompatibility [25]. Anjum et al. demonstrated the effectiveness of a NaCMC hydrogel containing antibiotic and anti-biofilm agents [26]. Here, we propose a strategy to inhibit biofilm formation by mixing the antibiotic rifampicin with an alginate hydrogel.

The purpose of this study is to develop a dual-drug-based scaffold that has a synergistic effect whereby biofilm formation is inhibited by RFP, which is an external drug, and bacterial activity is inhibited by CFZ, which is an internal drug that increases antibacterial activity. Consequently, a dual-drug-based scaffold that encapsulates a PCL-based scaffold containing CFZ and a scaffold with an RFP-loaded alginate hydrogel was successfully developed, and could potentially be a new strategy to target biofilms in osteomyelitis therapy.

## 2. Results

### 2.1. Characterization of the Dual-Drug-Based Scaffolds

**Figure 1:**
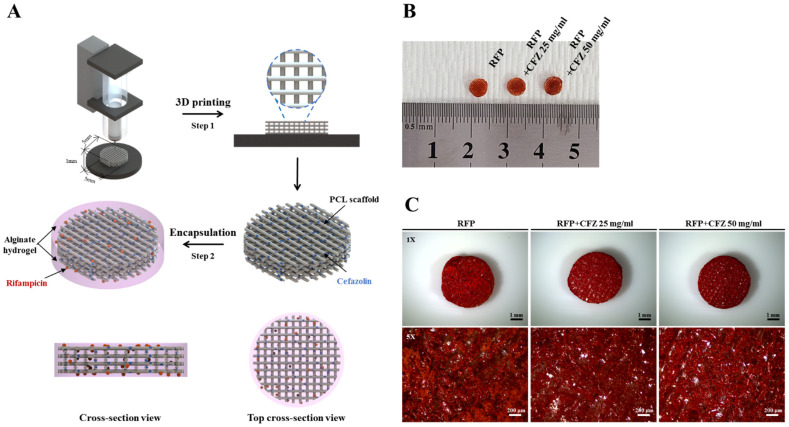
Fabrication procedure of the dual drug delivery systems: (**A**) 3D printing of a cefazolin–PCL scaffold to obtain a disk-shaped scaffold with 5 mm diameter and 1 mm thickness (step 1). Preparation of the cefazolin-loaded scaffold encapsulated in alginate mixed with rifampicin (step 2). (**B**) Gross view of the disk-shaped dual-drug scaffolds. (**C**) Microscope images showing the surfaces of rifampicin in the disk-shaped dual-drug scaffolds (1× bar = 1 mm; 4× bar = 200 μm).

To investigate the antibacterial activity of dual-drug-based scaffolds in vitro, we designed dual-drug-based scaffolds using two drugs (RFP: rifampicin; CFZ: cefazolin) at different sites. We hypothesized that in the dual drug delivery system, biofilm formation was inhibited by the external drug RFP, and the bacterial activity was suppressed by the internal drug CFZ, resulting in synergistically enhanced antibacterial activity. We combined 3D printing and the hydrogel encapsulation method (Figure 1A) to prepare a dual-drug-based scaffold to validate the antibacterial activity against *S. aureus*. The final dual-drug-based scaffold, with a disk-shaped design and a size of 5 mm, consisted of a PCL-based scaffold containing CFZ and an alginate hydrogel containing RFP that encapsulated the scaffold (Figure 1B). Microscopic images of the dual-drug-based scaffolds showed that the 4% alginate hydrogel containing RFP evenly surrounded the scaffold (Figure 1C). This suggests that the mixing of RFP and CFZ with alginate and PCL, respectively, and the compartmentalization of the dual-drug-based scaffolds were successful, and that the final product was able to inhibit biofilm formation and enhance antimicrobial activity.

### 2.2. Release of RFP and CFZ from the Dual-Drug-Based Scaffolds

**Figure 2:**
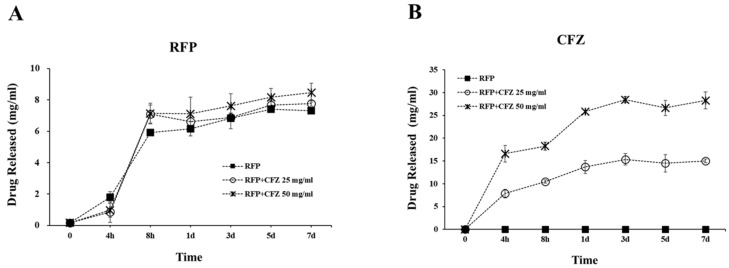
Cumulative drug release concentrations for the release of (**A**) RFP and (**B**) CFZ in PBS media. The drug release study was performed in PBS (pH 7.4), and the amount of drug release was estimated by HPLC analysis. RFP: rifampicin; CFZ: cefazolin.

As shown in Figure 2, RFP in the 4% alginate layer showed an abrupt release in the initial 8 h, and a continuous slow release for seven days. The accumulated RFP releases in the 4% alginate layer in seven days for the RFP only, RFP–CFZ 25 mg/mL, and RFP–CFZ 50 mg/mL groups were 7.63, 8.27, and 8.88 mg/mL, respectively. On the other hand, the CFZ release slowly increased during the seven days, reaching 0, 13.14, and 29.78 mg/mL in the PCL only, RFP–CFZ 25 mg/mL, and RFP–CFZ 50 mg/mL groups, respectively. This indicates that CFZ was encapsulated in the RFP–alginate layer, where the sudden release of CFZ could be well controlled. As for the surface layer, the RFP–alginate layer could suppress biofilm formation with its initial abrupt release. These results suggest that the stable combination of RFP and CFZ could exert prolonged antibacterial effects by allowing the continual release of CFZ via the encapsulation of RFP.

### 2.3. In Vitro Antibacterial Activity against S. Aureus

**Figure 3:**
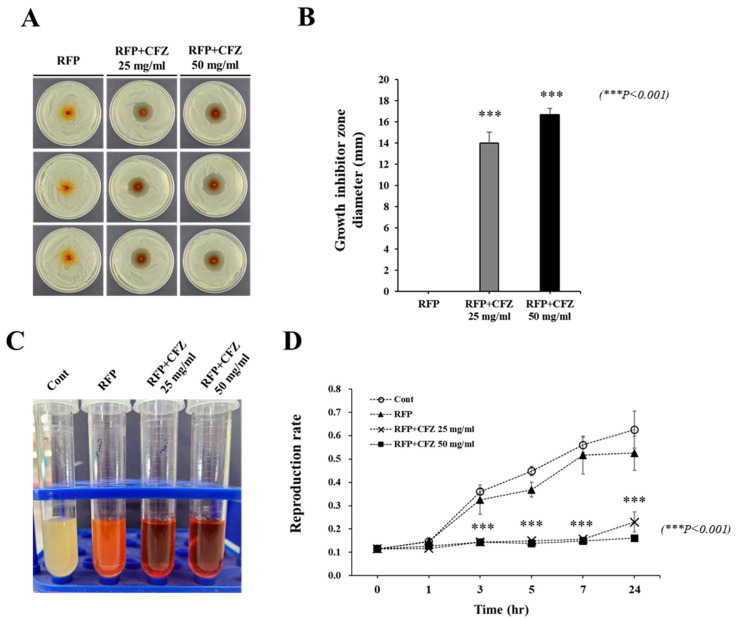
Antibacterial activity of the dual-drug-based scaffold: (**A**) Antibacterial activity of the dual drug-based scaffold in the disk diffusion assay. (**B**) Quantification of growth inhibition zone diameter. (**C**) Antibacterial activity of the dual-drug-based scaffold was assessed by broth dilution assay. (**D**) Bacterial count was monitored at 0, 1, 3, 5, 7 and 24 h after treatment with the dual-drug-based scaffold.

Figure 3 shows the antibacterial activity of the drug-based scaffolds against *S. aureus* after 24 h. In Figure 3A,B, the CFZ groups showed a diameter of inhibition of 14.00 ± 1.00 mm and 16.67 ± 0.58 mm at concentrations of 25 mg/mL and 50 mg/mL, respectively. However, the RFP-only group exhibited no inhibitory effects on *S. aureus* growth. The broth dilution assay showed no bacterial activity for the RFP–CFZ groups, indicating excellent antibacterial activity against *S. aureus*. However, the bacterial growth was prominent for both the PCL- and RFP-only groups, indicating low antibacterial activity. In addition, the reproduction rate of *S. aureus* was 0.63 ± 0.08 for the PCL-only group, 0.53 ± 0.07 for the RFP-only group, 0.23 ± 0.04 for the RFP–CFZ (25 mg/mL) group, and 0.63 ± 0.08 for the RFP–CFZ (50 mg/mL) group.

### 2.4. Anti-Biofilm Effect of Dual-Drug-Based Scaffolds

**Figure 4:**
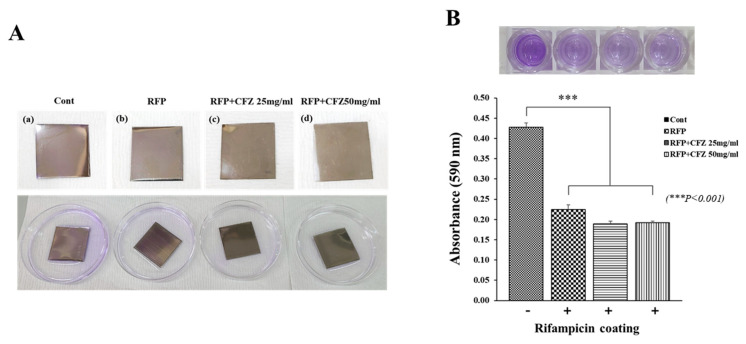
*Staphylococcus aureus* biofilm formation on stainless steel coupons: (**A**) Biofilm formation assays of *S. aureus* strains. Formed biofilm was stained with 0.1% crystal violet. (**B**) Quantification of *S. aureus* biofilm formation.

To observe the effect of the RFP–alginate layer on the formation of *S. aureus* biofilms, dual-drug-based scaffolds were added to the LB broth during biofilm growth, and the progression of biofilm formation within 48 h was monitored via the crystal violet assay (Figure 4). The crystal violet staining of *S. aureus* biofilms on stainless steel coupons submerged in LB broth is depicted in Figure 4A. The RFP–alginate layer gradually reduced biofilm formation to approximately 50% over 48 h, regardless of the presence of CFZ within the scaffolds. This was consistent with the absorbance measurement at 590 nm (Figure 4B), and together these results demonstrated that the presence of RFP significantly reduced *S. aureus* biofilm formation.

### 2.5. Analysis of Cell Viability

**Figure 5:**
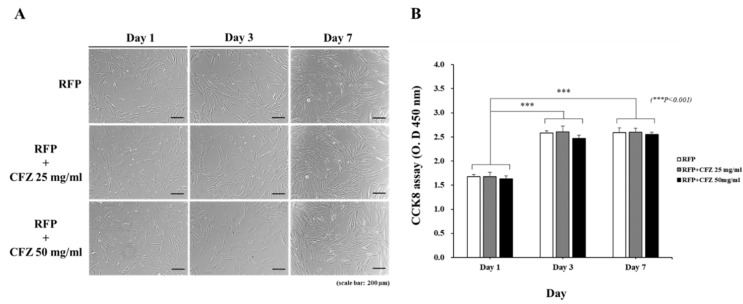
The cell viability of the dual drug-based scaffold; HOBs were cultured for 7 days: (**A**) Morphology of HOBs incubated with RFP, RFP + CFZ 25 mg/mL, and RFP + CFZ 50 mg/mL scaffold groups (×4); scale bar: 200 µm. (**B**) Effects of RFP, RFP + CFZ 25 mg/mL, and RFP + CFZ 50 mg/mL scaffold groups on HOB proliferation. HOB: human osteoblast cells.

To investigate the potential effects of the scaffolds on cell viability, we examined the proliferation of HOBs using the CCK8 assay, and evaluated cell morphology for seven days in each group. The proliferative activities of the cells on all scaffolds increased from day 1 to day 7 (Figure 5A,B). The scaffolds with and without RFP and CFZ exhibited relatively similar cell growth patterns. No significant differences were detected between the RFP-only and RFP–CFZ groups. This result clearly demonstrates that the scaffolds were not toxic to cells, and that the addition of the dual drug to the PCL scaffolds did not influence HOB growth in vitro.

## 3. Discussion

In this study, we developed a dual-drug-based 3D scaffold encapsulating a PCL-based scaffold loaded with CFZ and an alginate hydrogel containing RFP. Research on drug delivery systems aims at achieving improved therapeutic success for drugs, including those that have limited use because of toxicity, uneven distribution patterns, stability, and formulation difficulties. Until now, various types of polymeric drug delivery carriers have been reported, such as polymeric protein conjugates and polymers [27,28,29,30]. PCL is a semicrystalline, hydrophobic material that is compatible with a wide range of other polymer materials. Due to its polyvalent nature, fabrication ease, and good biocompatibility, it is established as a polymer of choice with a wide range of applications in targeted novel drug delivery and tissue engineering [31]. Hydrogels have been used extensively in the development of smart delivery systems. Alginates are natural polysaccharide polymers that can protect a drug from hostile environments (such as low pH and enzymes), while controlling drug release when the gel structure changes in response to the environment [32,33,34].

With this dual-drug-based scaffold, we demonstrated enhanced antibacterial activity, where biofilm formation was inhibited by RFP (an external drug), and bacterial activity was inhibited by CFZ (an internal drug). García-Alvarez et al. reported the fabrication of hierarchical 3D multidrug scaffolds with nanocomposite bioceramic and polyvinyl alcohol (PVA) prepared by 3D printing and an external coating of gelatin–glutaraldehyde (Gel–Glu) [35]. However, a multidrug scaffold using heat-sensitive antibiotics for osteomyelitis treatment has not yet been investigated. CFZ is an antibiotic commonly used for osteomyelitis, and has been proven to be effective against methicillin-susceptible *Staphylococcus aureus* and non-resistant *streptococcus*, but is considered a poor antibacterial agent due to its heat sensitivity [16,36]. This study was the first to develop a dual-drug-based scaffold using a 3D printing system capable of low-temperature processing that could maintain the antibacterial activity of CFZ (Figure 1).

Our data confirmed that RFP contained in the alginate of the outer layer was released explosively for up to 8 h, whereas CFZ mixed in the PCL on the inside was continuously released for up to 7 days in proportion to the concentration. We demonstrated that the dual-drug-based scaffold maintained the antibacterial effects of antibiotics (Figure 3). These data suggest that RFP contained in the alginate of the outer layer that was released explosively at the beginning prevented biofilm formation, and the stably released CFZ in the PCL scaffold increased the antibacterial activity, thereby realizing the synergistic effect of the dual drug delivery system (Figure 2). 

We previously reported that a rifampicin-loaded scaffold with melted PCL could be fabricated by 3D printing at a low temperature, and we demonstrated that heat-labile antibiotics could be a possible osteomyelitis treatment via enhanced antibacterial activity [12]. We confirmed the RFP’s inhibition of biofilm formation by demonstrating the antibacterial activity of the RFP-loaded alginate hydrogel (Figure 4). Although this antibacterial activity was confirmed in the previous paper [12], it was not evident in this report (Figure 3). We suspect that the time difference between the stable release from the 3D-printed scaffold and the explosive release after mixing with the alginate contributed to this.

We performed in vitro experiments using human osteoblast (HOB) cells to investigate whether the dual-drug-based scaffold affected cell proliferation. Duewelhenke et al. investigated the effects of 20 different osteomyelitis-related antibiotics on primary human osteoblasts (PHOs), the osteosarcoma cell line MG63, and the epithelial cell line HeLa [37]. While that study reported that high concentrations of CFZ and RFP decreased cell proliferation and metabolic activity, we did not observe any influences on HOB growth in vitro by the loading of CFZ and RFP into the dual-drug-based scaffold (Figure 5). This may be explained by differences in energy metabolism between the different cell lines. The study’s limitations that should be addressed in future research approach include the drug release test samples, which could offer a release kinetics model for their innovative system, and the fact that the inflammatory and toxicity tests would be further employed through the osteomyelitis animal model.

## 4. Conclusions

In this study, we developed a dual-drug-based 3D scaffold containing RFP and CFZ, which was manufactured by 3D printing using the antibiotic-mixed PCL with an external coating of antibiotic-loaded alginate. These dual-drug-based scaffolds, which were characterized by an early burst of RFP release followed by a continuous and prolonged release of CFZ, were able to inhibit biofilm formation and suppress *S. aureus* growth within short time periods. Therefore, we believe that our dual-drug-based scaffold represents new opportunities to improve osteomyelitis therapy.

## 5. Material and Methods

### 5.1. Fabrication of Rifampicin- and Cefazolin-Loaded Dual-Drug Scaffolds

To fabricate the dual-drug-based scaffolds, a drug-loaded scaffold was first prepared by 3D printing, as reported previously [12]. Briefly, PCL (MW: 45,000; Sigma-Aldrich, St Louis, MO, USA) and cefazolin (CFZ) (Chong Kun Dang pharmaceutical Corp., Seoul, Korea) were mixed at concentrations of 25 mg/mL and 50 mg/mL, respectively, and then ejected using a 3D bioprinting system (Geo Technology, Incheon, Korea) to a 200 μm nozzle (inner diameter) supplied with 800 kPa pressure at a constant speed of 100 mm/min. The resultant cefazolin-loaded scaffolds were cut into 5 mm disks using a punch and immersed in another 12-well plate containing 4% sodium alginate solution (Sigma-Aldrich, St Louis, MO, USA) blended with 9 wt% rifampicin (RFP) (Tokyo Chemical Industry Co., Ltd., Tokyo, Japan), and 100 mM calcium chloride solution (Sigma-Aldrich, St Louis, MO, USA) was added for 30 s at room temperature to encapsulate alginate. The fabrication process of the dual-drug-based scaffold is described in Figure 1A.

### 5.2. In Vitro Dual Drug Release

A dual drug-based scaffold was dipped in a 5 mL tube, and 4 mL of phosphate-buffered saline (PBS, pH 7.4; Gibco, Waltham, MA, USA) solution was added to the tube. The tube was placed in a constant temperature incubator for seven days. The incubator was set to 37 °C, and 1 mL samples were extracted intermittently from the release medium, before 1 mL of fresh medium was added to the release medium. The collected supernatants were measured using UV spectrophotometry and HPLC analysis. The amount of RFP released was verified by measuring the absorbance at 340 nm wavelength using UV spectrophotometric analysis, and the released CFZ was measured using a SHISEIDO SI-2 (Osaka soda, OSK, Japan) HPLC with a SHISEIDO dual pump, 3004 column oven, and 3002 detectors set at a wavelength of 272 nm.

### 5.3. Antibacterial Activity Assay

The antibacterial activity of the dual-drug-based scaffold was characterized by soft agar and liquid tests to study bacterial growth inhibition and bactericidal action, respectively. Gram-positive *S. aureus* (KCTC No. 3881) was cultured in Luria–Bertani (LB) broth (BD, Franklin Lakes, NJ, USA) at 37 °C overnight until the optical density, which represented the concentration of bacteria reaching approximately 1.5 × 10^8^ colony-forming units per ml (CFUs/mL). In accordance with ISO standard 17,025 for measuring bacterial zones of inhibition, 100 μL of bacterial culture was added to LB agar (BD, Franklin Lakes, NJ, USA). The samples were then added to the plates for testing. Zones of inhibition were measured for the fabricated antibiotic test groups after 24 h of incubation at 37 °C. Each sample group was tested on three plates for reliability and reproducibility. LB broths were inoculated with 50 μL of bacterial culture and treated with specimens from each group. The cultures were incubated for 24 h at 37 °C and 170 rpm on a shaker. Triplicates from each group were tested and compared with controls. The absorbance at 600 nm wavelength was measured using an enzyme-linked immunosorbent assay (ELISA) reader (Soft Max Pro5, Molecular Devices, San Jose, CA, USA). Each sample group was tested on three plates for reliability and reproducibility.

### 5.4. Biofilm Formation Assay

An *S. aureus* strain was selected for biofilm formation studies on stainless steel coupons and grown overnight in LB broth at 37 °C and 150 rpm. Next, 500 µL of *S. aureus* was inoculated into 25 mL of LB broth in a tube, into which stainless steel coupons (AISI 201, 1.5 × 1.5 cm^2^) were immersed. Dual-drug-based scaffolds were then added to each tube. The stainless steel coupons were incubated at 37 °C for 48 h and then washed three times with sterile distilled water and dried. After drying, the stainless steel coupons were stained with 1 mL of 0.5% crystal violet (CV) solution (Sigma-Aldrich, St Louis, MO, USA). The dye bound to the biofilm was solubilized with 1 mL of 99% ethanol (Samchun Chemicals, Gyeonggi-do, Korea), and the absorbance was determined at 595 nm [38].

### 5.5. Cell Proliferation Assay

Cell culture and cell proliferation assays were performed as previously reported [12]. Human osteoblasts (HOBs) were seeded at a density of 3 × 10^5^ cells per well in a 48-well plate. Dual-drug-based scaffolds were then added to each well. After 1, 3, and 7 days, 100 µL of CCK8 solution (Dojindo, Kumamoto, Kyushu, Japan) was added to each well, and the cells were incubated at 37 °C for 2 h. Absorbance at 450 nm was measured using an ELISA reader.

### 5.6. Statistical Analysis

All experiments were repeated three times, and the average values are presented unless otherwise stated. Data are presented as the mean ± standard deviation. The statistical significance of the results was determined using ANOVA. For all experiments, significance was defined as * *p* < 0.05, ** *p* < 0.01, and *** *p* < 0.001.

## Data Availability

All data used in this paper are contained within the article.
